# Livestock management, beaver, and climate influences on riparian vegetation in a semi-arid landscape

**DOI:** 10.1371/journal.pone.0208928

**Published:** 2018-12-11

**Authors:** Kurt A. Fesenmyer, Daniel C. Dauwalter, Carol Evans, Todd Allai

**Affiliations:** 1 Trout Unlimited, Boise, Idaho, United States of America; 2 US Bureau of Land Management, Elko, Nevada, United States of America; 3 US Bureau of Land Management, Vale, Oregon, United States of America; Universitat Autonoma de Barcelona, SPAIN

## Abstract

Riparian and aquatic habitats support biodiversity and key environmental processes in semi-arid and arid landscapes, but stressors such as conventional livestock grazing, wildfire, and drought can degrade their condition. To enhance habitat for fish and wildlife and increase resiliency in these critical areas, land managers in the interior western United States increasingly use alternative grazing strategies, beaver management, or beaver dam surrogates as low-effort, low-expense restoration approaches. In this study we used historical archives of satellite and aerial imagery spanning three decades to characterize riparian vegetation productivity and document beaver dam occurrences, then evaluated vegetation productivity relative to land management associated with livestock grazing and beaver dam densities while accounting for climate and wildfire. After controlling for stream characteristics such as stream size, elevation, and stream slope, we demonstrate a positive response of riparian area vegetation to conservation-oriented grazing approaches and livestock exclosures, extensive beaver dam development, increased precipitation, and lack of wildfire. We show that livestock management which emphasizes riparian recovery objectives can be an important precursor to beaver activity and describe 11–39% increases in floodplain vegetation productivity where conservation-oriented grazing approaches or livestock exclosures and high beaver activity occur together on low-gradient sites. Land management decisions can therefore potentially confer resiliency to riparian areas under changing and variable climate conditions–the increased vegetation productivity resulting from conservation-oriented grazing or exclosures and high amounts of beaver activity at our sites is the equivalent to moving conventionally-grazed, low-gradient sites without beaver up at least 250 m in elevation or increasing water year precipitation by at least 250 mm.

## Introduction

Riparian and aquatic habitats within stream corridors support a diverse array of fish and wildlife taxa and environmental processes [1, 2]. The importance of riparian and aquatic habitats for biodiversity is often disproportionate to their overall area, especially in arid and semi-arid ecosystems where the narrow ribbon of productivity in and along streams stands in stark contrast to xeric uplands. In these systems, isolated and scarce riparian and aquatic habitats can provide seasonal or year-round habitat for a majority of terrestrial [3] or avian [4] taxa and host endemic and imperiled aquatic taxa [5, 6].

The use of riparian and freshwater habitats in drylands by fish and wildlife taxa is associated with specific attributes of those habitats. For example, in the Great Basin of the interior western United States, greater sage-grouse (*Centrocercus urophasianus*) use of riparian systems during the brood-rearing period requires the presence of high-quality forage of forbs, succulent vegetation, and insects [7, 8]. Trout of the interior western US (*Oncorhynchus* spp.) have been linked to cool stream temperatures [9–11] and the presence of woody streamside vegetation [11, 12]. Many of the aquatic and riparian habitat conditions required by fish and wildlife can be degraded by the individual and synergistic effects of disturbances in dryland ecosystems, which include wildfire [8,13–16], drought [16–18], flooding [13, 19], and livestock grazing [20, 21].

Controls on discrete wildfire, flood, and drought events in dryland ecosystems occur at regional or global scales. For example, drought and flooding in drylands can be associated with global climate variability, such as the El Niño-Southern Oscillation [22, 23], while wildfire can be linked to regional climate [24, 25] and vegetation dynamics, including invasion of non-native grasses [26, 27]. In contrast, livestock grazing is controlled at local scales through the decisions of land managers and potentially represents a chronic disturbance, especially where concepts of managed grazing are not specifically implemented [28].

Livestock can alter the amount, structure, and community composition of vegetation within riparian areas through consumption, selection, and trampling. These alterations to vegetation remove important drivers of stream morphology, from channel-forming large wood to bank-stabilizing roots, while the compaction and shear of livestock hooves alters the physical form of floodplains [20, 21] and reduces riparian habitat quality for wildlife [29, 30]. For instream habitats, unmanaged livestock grazing may be associated with decreased habitat quality for aquatic taxa in the form of decreased streambank stability, higher concentrations of fine sediments, decreased riparian vegetation, and altered food webs [20, 31–34]. The legacy effects of livestock grazing in riparian systems can persist long after livestock use has ended [35].

To enhance the resistance and resiliency of streams and riparian areas to wildfire and drought and in response to known impacts of livestock, land managers increasingly use conservation-oriented grazing strategies to mitigate impacts to vegetation, biodiversity, and hydrology within the stream corridor and to restore proper riparian function [36]. These strategies attempt to balance grazing periods with opportunities for plant growth by adjusting grazing season, duration, and intensity, and often include a combination of tools and techniques designed to improve livestock distribution and reduce use of riparian areas [28, 36]. Examples of practices that promote rather than impede recovery include altering the timing of cattle use to outside the growing season (i.e., spring or fall), limiting duration or intensity of use within seasons, resting pastures during some years, using rotation among pastures, and excluding some areas from use completely. Common examples of tools and techniques which improve livestock distribution and management include offsite water developments, fencing, use of supplements such as salt blocks, and implementing herd management and animal husbandry practices such as herding and riding, culling, and changing class of livestock [28, 36].

Concurrently, in dryland ecosystems within the native distribution of North American beaver (*Castor canadensis*) there is increasing awareness among land managers of the role of beaver, beaver dams, and beaver dam analogues as restoration tools in degraded riparian systems [37–40]. In arid and semi-arid landscapes, beaver dams increase water storage in streams and in adjacent floodplains, maintain water within the river course, and promote riparian vegetation [41]. Active and abandoned dams are associated with elevated structural and thermal heterogeneity of instream habits [42–46] and increased woody vegetation in riparian habitats [47]. Gibson and Olden [41] provide a thorough review of the response of hydrology, geomorphology, water quality, riparian vegetation, and wildlife to the habitat manipulations of beaver.

As land managers implement conservation grazing strategies and beaver restoration approaches, monitoring how habitats are affected by these treatments can be challenged by the long time horizon and variability in response. Archives of remote sensing data provide an opportunity to efficiently and effectively monitor the response of riparian vegetation to disturbances and management treatments over decadal time horizons [48, 49]. Satellite and aerial imagery are well-suited for monitoring riparian vegetation productivity and trend in dryland ecosystems due to the quality and time series of remotely-sensed data products, the seasonal and structural contrast between riparian areas and adjacent uplands, and the logistical challenges of field monitoring in undeveloped landscapes [50]. Satellite-derived measures of vegetation productivity, such as the normalized difference vegetation index (NDVI), use multispectral information to estimate the amount and vigor of green vegetation [51]. Higher resolution aerial imagery (as captured by planes or drones) has been used for identifying specific features of riparian areas, such as dewatered channels [52], density of vegetation [53], and individual beaver dams [54].

In this study, we used remote sensing datasets to evaluate changes in riparian productivity in five semi-arid watersheds due to changes in livestock grazing regimes, while also accounting for the environmental setting created by stream characteristics as well as other ecosystem changes due to beaver colonization, wildfire, and climate variability. Our objectives were to 1) assemble a unique dataset of vegetation productivity, digitized beaver dam locations, and grazing histories spanning three decades; 2) apply a linear mixed modelling approach to evaluate biotic and abiotic predictors of riparian vegetation productivity and quantify anticipated gains in productivity in response to management decisions; and 3) use path analysis to evaluate direct and indirect linkages between biotic and abiotic factors and describe the restoration pathways of riparian vegetation in response to management strategies. Our study uses extensive archives of satellite and aerial imagery and leverages new developments in cloud-based remote sensing to facilitate rapid analysis of long-term datasets.

## Methods

### Study area

**Fig 1:**
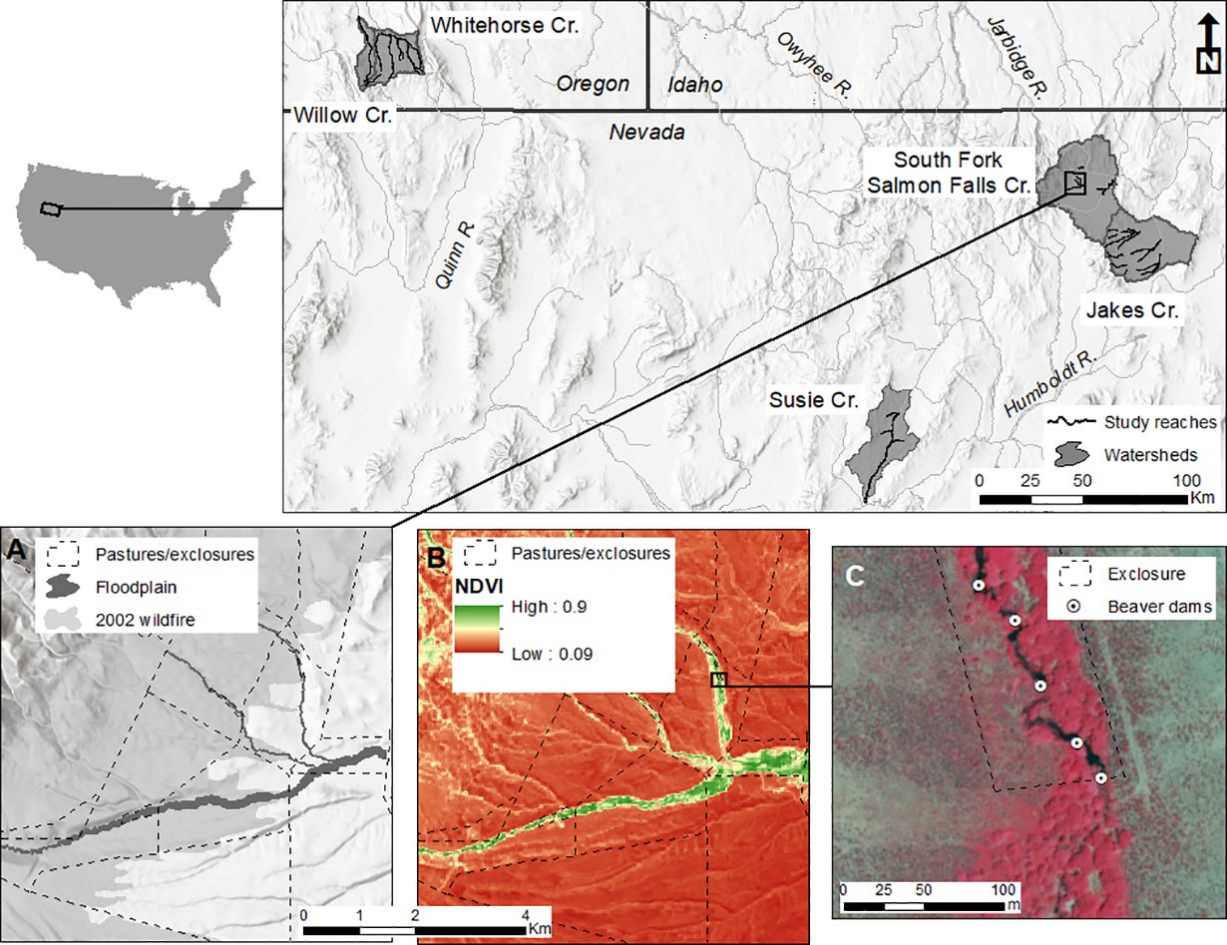
Locator map, observation units, and imagery examples. Top panels: Location of study watersheds. Panel A: Example of observation units, delineated by pasture or exclosure, modeled floodplain boundaries, and individual streams, with example wildfire boundary overlaid. Panel B: Example maximum growing season NDVI measured from Landsat 8 in 2015. Panel C: Example beaver dam locations identified in 2015 false-color National Agriculture Imagery Program (NAIP) imagery within an exclosure and adjacent pasture area. Landsat imagery is reprinted from US Geological Survey and NAIP imagery is reprinted from USDA Farm Service Agency, both under a CC BY license (public domain).

Our analysis area includes Susie Creek (116°W, 41°N), Jakes Creek (115°W, 41°30’N), and South Fork Salmon Falls Creek (115°W, 41°45’N) in Nevada and Willow Creek (118°15’W, 42°10’N) and Whitehorse Creek (118°W, 42°10’N) in Oregon (Fig 1). All watersheds are in the Northern and Central Basin and Range ecoregions in the western United States [55] and within the floristic Great Basin (hereafter Great Basin). Climate at all sites is characterized by hot, dry summers and cold, moist winters [55] which results in hydrologic regimes with peak flows associated snowmelt and rain in spring and low baseflow during summer [56]. Average elevation ranges from 1,750 m in Willow Creek to 2,100 m in South Fork Salmon Falls Creek. Vegetation along streams and on mesic sites is dominated by native taxa and includes aspen (*Populus tremuloides*), black cottonwood (*Populus balsamifera*), willow (*Salix* spp.), and thinleaf alder (*Alnus incana*) at higher elevations and a variety of willows, sedges (*Carex* spp.), rushes (*Scirpus* and *Eleocharis* spp.), and riparian grasses and forbs at lower elevations. Sagebrush (*Artemisia tridentata*) and a variety of perennial bunchgrass species dominate the uplands. All sites provide riparian or aquatic habitat for at least one imperiled or sensitive taxa, including greater sage-grouse, Great Basin distinct population segment of Columbia spotted frog (*Rana luteiventris*), Lahontan cutthroat trout (*Oncorhynchus clarkii henshawi*), and Columbia River redband trout (*Oncorhynchus mykiss gairdneri*).

Land managers in each watershed have implemented conservation-oriented grazing management at various times during recent decades with an objective to improve riparian conditions and habitat for imperiled or sensitive fish and wildlife. Each watershed contains rangelands administered by US Bureau of Land Management (BLM), US Forest Service, and private landowners within grazing allotments comprised of pastures and exclosures. Livestock management plans developed for allotments define seasons of use, stocking rates, and pasture and exclosure boundaries, and often include specific riparian habitat goals. In some cases, managers implemented temporary closures or restrictions in grazing use to mitigate the effects of wildfire. Beaver have expanded their habitat use in each watershed over the last thirty years through natural colonization and without the aid of any specific wildlife management strategies (e.g., trapping limits or translocation).

Wildfires and climate variability have affected the study watersheds over the three decades: five wildfires have affected riparian areas in the watersheds, and water year precipitation has varied up to 125% from year to year (S1 Dataset). Of the five watersheds in the study, only Susie Creek includes a streamflow gage. There, peak water year flows from 1993–2015 ranged from 0.34–45 cms and mean water year flows ranged from 0.014–0.97 cms (S1 Dataset); these streamflow metrics moderately correlate with water year precipitation (Pearson correlation coefficient = 0.70 and 0.61, respectively).

### Observation unit delineation

In each study watershed we created observation units (analogous to sites) based on the riparian area of individual streams within pasture or exclosure boundaries. Within each drainage, we delineated riparian areas for named streams in the National Hydrography Dataset (NHD; [57]) using the Valley Bottom Extraction Tool (V-BET; [58]) and a 10-m resolution digital elevation model [59] using a GIS software (ArcGIS 10.5—ESRI, Inc.). The V-BET tool identifies a variable-width floodplain based on stream size attributes and terrain characteristics such as slope. We then intersected the riparian area with the local catchment area of each stream reach and pasture or exclosure boundaries [60, 61] to create our final observation units. The final units represent the floodplain area of each unique combination of named stream, Strahler stream order, and pasture or exclosures (e.g., the 2^nd^ order section of Doolittle Creek in the 15 Mile pasture). These units allowed us to characterize the effect of grazing treatments on riparian vegetation within observation units, while controlling for environmental variability within pastures or exclosures associated with stream characteristics (e.g., streamflow, slope, elevation).

### Riparian vegetation productivity

We used a 31-year historical archive of seasonal peak NDVI measurements from Landsat satellite imagery as a response variable and estimate of annual riparian vegetation productivity. NDVI is a remotely-sensed measure of active photosynthesis and vegetation productivity widely used to track vegetation dynamics in response to environmental and land management changes [48, 62, 63]. NDVI is commonly used as a proxy for vegetation condition as related to factors such as leaf area, vegetation cover, and net primary productivity [64].

We generated annual maps of maximum-value composite NDVI for the late growing season (late July–mid-September) from US Geological Survey Landsat 5, 7, and 8 Collection 1 Tier 1 surface reflectance products ([65]; 30 m^2^ pixel resolution, typically 6 Landsat scenes per late growing season) using Google Earth Engine [66], and summarized the average value within each observation unit for each year from 1985–2015. We used a maximum-value composite (“greenest pixel”) approach to minimize phenological differences across observation units [62] and focused on late growing season NDVI to minimize the influence of annual grasses and other vegetation outside of the riparian area, which senesce by mid-summer [67]. We also calculated an annual measure of the proportion of each observation unit with at least one valid NDVI pixel value (i.e., not identified as cloud cover, shadow, snow, or water in Landsat Collection 1 Pixel QA band attributes) for the late growing season and excluded any observation unit with an average value less than 0.5. For Landsat 8 NDVI data, we applied the function described by Roy and others [68] to adjust for comparison with Landsat 5 and 7.

### Spatial covariates

We selected a set of spatial covariates for predicting riparian vegetation productivity in observation units based on a literature review of influences on riparian vegetation and the availability of datasets for the study areas. The final list of covariates includes a mix of continuous and categorical datasets, observation unit-scale and watershed scale-data, and data with and without a temporal component (Table 1). For predicting annual riparian vegetation productivity in observation units, we summarized covariates for each observation unit for each year. Where observation unit-scale data were lacking, we applied watershed-scale data to all observation units in the watershed; where temporal data were lacking, we applied an identical value to all observation units for all years.

**Table 1:** Spatial covariates used to predict riparian vegetation productivity.

Variable	Definition	Temporal and spatial scale	Data source
Livestock grazing regime	Categorical measure of livestock use (conservation, exclosure, or conventional); classified as "Conservation" if listed in Swanson et al. 2015, Table 2 as supporting riparian functions and allowing recovery	Category by year (1985–2015) for each observation unit	BLM [60, 61]
Beaver dams	Continuous measure of beaver use of stream as dams per stream km	Density by year for each observation unit; limited availability, see Table 3	See Table 3
Wildfire	Categorical measure of wildfire or wildfire recovery (unburned, burned); classified as "Burned" for 2 years following wildfire if at least 1/3 of observation unit overlaps with wildfire boundary	Category by year (1985–2015) for each observation unit	MTBS Burned Areas Boundaries Dataset [69]
Precipitation	Continuous measure of total water year precipitation in dm	Sum of daily precipitation from Oct 1 of previous year to Sept 30 for each year (1985–2015) by watershed; same value applied to all observation units in watershed	Daymet V2 [70] (1 km^2^ resolution)
Drought	12—month Evaporative Demand Drought Index (EDDI)	12-month EDDI for Aug 1 for each year (1985–2015) by watershed; same value applied to all observation units in watershed	EDDI Map Archive [71] (12 km^2^ resolution)
Stream size	Continuous measure of upstream contributing watershed area in km^2^	Value for each observation unit; not temporal–same value applied to all years	NHD-Plus [72]
Stream slope	Continuous measure of stream slope as %	Value for each observation unit; not temporal–same value applied to all years	Max.—min. elevation on stream from 10-m DEM [59]/stream length from NHD [57]
Elevation	Continuous measure of average stream elevation in km	Value for each observation unit; not temporal–same value applied to all years	10-m DEM [59]

Livestock grazing alters the amount, structure, and community composition of riparian vegetation [20], while conservation-oriented grazing regimes and livestock exclosures can facilitate riparian vegetation recovery [28]. We compiled grazing histories dating to at least 1985 (the first year with satellite imagery at 30 m^2^ resolution) for all observation units based on BLM records and grazing plans. We characterized grazing regime for each unit by year as “conservation-oriented,” “exclosure,” or “conventional.” Conservation-oriented grazing regimes support riparian function and allow recovery after use and include strategies such as short grazing periods, long post-grazing recovery periods, periods of rest, and explicit riparian objectives [28]. Exclosures are fenced areas built to exclude livestock completely and include both reference exclosures (linear features on low-gradient stream channels in alluvial basins) and protective exclosures (features focused on areas of specific concern, including springs) [73]. Although we consider exclosures to be a conservation strategy, these areas were considered separately from conservation-oriented grazing treatments. Conventional livestock grazing practices include grazing throughout the growing period with little or no effort to control amount, duration, or distribution of livestock use in specific areas [36] and tend to preclude recovery of riparian areas. Land managers largely initiated the switch from conventional grazing to conservation-oriented grazing in 1985 and 1996 in South Fork Salmon Falls Creek, in 1989 in Willow and Whitehorse Creeks, in 1992 and 2007 in Susie Creek, and in 2001 in Jakes Creek, although timing varied by pasture. Grazing strategies for each watershed are described in more detail in existing literature [28, 74–76].

Beaver can create increased hydrological connectivity and water storage in floodplains through dam construction, which can have a positive effect on floodplain vegetation productivity [41]. Despite consuming and cutting vegetation, beaver can promote riparian vegetation biomass and vigor [77]. To characterize the density of beaver dams within observation units, we digitized the locations of individual dams visible within aerial photographs. Source imagery included single frame historical true-color or false-color aerial photos from BLM or US Geological Survey Earth Explorer archives which required georeferencing, and more contemporary digital, georeferenced black and white (digital ortho-quadrangles) and 3- and 4-band National Agricultural Imagery Program images (Table 2). All imagery had a spatial resolution of 0.4–1 m^2^. We used a consistent map scale for digitizing: 1:2,500 for the finer resolution imagery and 1:5,000 for the coarser resolution imagery. We are unable to determine through aerial imagery interpretation if beaver dams are active or abandoned, and therefore cannot estimate the degree to which sites are actively “managed” by beaver through herbivory, dam construction and repair on an annual basis. Nonetheless, riparian vegetation is likely benefiting from the presence of beaver dams where beaver are absent by virtue of floodplain saturation and water storage [41].

**Table 2:** Imagery used to identify beaver dams.

Year	Willow	Whitehorse	SF Salmon Falls	Jakes	Susie	Imagery resolution (m^2^)
1986			BLM CIR (3)			0.1
1991					BLM color (0)	0.1
1994	DOQ B&W (0)	DOQ B&W (9)	DOQ B&W (14)	DOQ B&W, BLM color (18)	DOQ B&W (0)	1
1998					BLM CIR (25)	0.4
2000	DOQ B&W (14)	DOQ B&W (11)				1
2005	NAIP 3-b (25)	NAIP 3-b (25)				0.5
2006			NAIP 3-b (23)	NAIP 3-b (45)	*NAIP 3-b (32)*	1
2009	NAIP 4-b (88)	NAIP 4-b (42)				0.5
2010			NAIP 4-b (46)	NAIP 4-b (32)	NAIP 4-b (137)	1
2011	NAIP 4-b (42)	NAIP 4-b (33)				1
2013			NAIP 4-b (104)	NAIP 4-b (42)	NAIP 4-b (156)	1
2014	*NAIP 4-b (37)*	*NAIP 4-b (39)*				1
2015			NAIP 4-b (176)	NAIP 4-b (57)	NAIP 4-b (245)	1

BLM CIR = Bureau of Land Management low elevation color infrared imagery; BLM color = Bureau of Land Management low elevation color imagery; DOQ B&W = Black and white digital orthophoto quadrangle; NAIP 3-b = National Agriculture Imagery Program true color imagery; NAIP 4-b = true color imagery with infrared band. Count of beaver dams observed in imagery in parenthesis. Italics indicate imagery which capture a wildfire recovery period.

Wildfire can consume riparian vegetation [78]; to characterize fire effects within observation units, we calculated the proportion of each observation unit which overlapped with a wildfire identified in the Burned Areas Boundaries Dataset [69] for each year from 1985–2015. We categorized an observation unit as burned and recovering ("Burned") for two years if at least one-third of an observation unit overlapped with a wildfire boundary. We selected a two-year recovery period based on preliminary data exploration for our sites; riparian vegetation in Great Basin sites can rapidly recover from wildfire through basal sprouting and recruitment of seeds and vegetative propagules distributed by streamflow [78]. We determined which two years received the “Burned” designation based on the satellite imagery: if the satellite record for the late growing season included a pre-fire observation, the observation unit was considered “Burned” for the two years following the year of the fire; if the satellite record did not include a pre-fire observation, then the observation unit was considered “Burned” for the year of the fire and following year. We characterized all other years as “Unburned.”

Annual growth and vigor of riparian vegetation is linked to annual precipitation [18, 48]. Annual precipitation increases riparian water availability through higher soil moisture and drives many key seasonal streamflow characteristics (peak flow, low flow, base flow), which affect riparian vegetation growth, dispersal, and structure [1, 79–82]. To account for annual climate variability related to precipitation, we summed daily precipitation values in the Daymet V2 dataset [70] for each water year (October 1 of the prior year through September 30 of year of interest) for each year from 1985–2015 at the geometric centroid of each watershed. We used water year to capture fall and winter snowfall which does not contribute to streamflow until the following spring. Due to the lack of streamflow gage data for each study watershed, we used water year precipitation as a proxy for streamflow; peak water year streamflow and mean water year streamflow are moderately correlated with water year precipitation in Susie Creek (Pearson correlation coefficient = 0.70 and 0.61, respectively, for 1993–2015), the only study watershed with a streamflow gage.

Sustained drought affects vegetation productivity by amplifying soil moisture supply limitations through atmospheric water demand [49, 83]. To account for climate variability related to drought, we summarized the Evaporative Demand Drought Index (EDDI) for a 12-month period ending Aug 1 for each year from 1985–2015 at the geometric centroid of each watershed. EDDI accounts for moisture, wind speed, solar radiation, and temperature on the land surface and in the atmosphere to characterize sustained and acute periods of drought [83, 84]; positive EDDI values indicate periods of high evaporative demand and potential for drought. The 12-month EDDI reveals sustained drought stress; while other time scales of precipitation or EDDI may be better predictors of NDVI, comprehensive evaluation of different periods was beyond the scope of this analysis.

We calculated a set of static covariates (i.e., those lacking a temporal component) related to stream characteristics within observation units, including stream size, stream slope, and elevation, using NHD-Plus [75], NHD [60], and a 10-m digital elevation model [62]. The stream size covariate captures within-watershed variability of streamflow when considered with precipitation in absence of reach-scale streamflow data [85]. Seasonal flows affect riparian vegetation growth, dispersal, and structure [1, 79–82]. Stream slope determines floodplain form (e.g., depositional vs. erosional environments) and thus riparian vegetation potential [1, 79, 80, 86]. The elevation covariate captures variability of vegetation communities and productivity along elevation gradients [79] and serves to account for the variability of watershed-scale precipitation within observation units.

### Statistical analysis

We evaluated the influence of grazing regime and beaver colonization on riparian vegetation productivity while accounting for other important temporal and spatial factors using linear mixed models (LMM). We fit several candidate LMMs, which are essentially multiple regression models with a random effect, to the data and evaluated them in a model selection framework. We used average peak annual NDVI within observation units as a response variable, and evaluated grazing regime, beaver dam density, water year precipitation, drought, wildfire, elevation, stream size, and stream slope as predictor variables. We used pasture by watershed as a random effect and grazing management (categorical: Conventional, Conservation, Exclosure) and wildfire (Unburned = 0; Burned = 1) as treatments (fixed effects). Candidate models were constructed of all combinations of predictor variables with the constraint that EDDI and precipitation could not be in the same model due to their correlation (r = -0.604). We hypothesized positive relationships between NDVI and beaver dam density, precipitation, elevation, and stream size due to increased water availability. We hypothesized negative relationships between NDVI and conventional grazing, EDDI, wildfire, and stream slope due to removal of plant biomass by grazers and wildfire, decreased water availability for vegetation under drought conditions, and poorly defined and highly dynamic floodplains along higher gradient streams. We also evaluated the interaction of slope and beaver dam density due to the potential differences in the effect of beaver on vegetation productivity in high vs. low slopes due to increased impoundment size and floodplain connectivity in low slope areas [87], as well as the interaction of grazing regime and beaver dam density due to the potential competition between cattle and beaver for woody vegetation forage [88]. Candidate models were constructed using all combinations of variables and fit to the data, and model plausibility was determined using Akaike’s Information Criterion for small sample size [89]. Candidate models within 4 AICc units of the best model (minimum AICc) were considered plausible. Fit of the most plausible regression model was evaluated using both the marginal and conditional R^2^ [90].

We also used path analysis as a complement to the linear mixed model analysis to evaluate the association of riparian vegetation productivity with grazing regime, beaver dam density, wildfire, climate, and stream characteristics, as well as test for explicit linkages between those factors. Path analysis is an extension of multiple regression that estimates the magnitude and significance of direct and indirect relationships between sets of observed variables while accounting for their covariance, and it produces a directed graph (path diagram or model) that shows these inter-relationships [91]. Path analysis is often thought of as more confirmatory of causal relationships than other statistical modelling approaches [92]. We developed a conceptual model of the relationships among significant predictor variables identified in the LMM to inform our initial path analysis. For the path analysis, we reconfigured grazing regime into a continuous variable that was the sum of the main and interaction parameter estimates from the multiple regression model (Conventional = 0; Conservation = 0.030; Exclosure = 0.043). This allowed us to keep the path analysis simple instead of duplicating relationships associated with two separate factors (i.e., as Typical = 0, Conservation = 1; and Conventional = 0 and Exclosure = 1) when the general relationships were expected to be identical. In addition to direct linkages between each predictor variable and NDVI, the model also includes a hypothesized link between beaver dams and precipitation and stream characteristics such as stream size, slope, and elevation to reflect geomorphic and streamflow controls affecting beaver dam placement and permanence [87]; between precipitation and wildfire to reflect the association between drought years and fire [24, 25]; and between grazing regime and beaver dam density reflecting the potential for conventional grazing to limit woody vegetation, a key source of dam construction materials and food for beaver [77, 88]. The initial path model was fit using the lavaan package in R (R Core Team 2015; [93]), and the semPaths function was used to display the directed graph [94]. We used standardized coefficients to examine the significance of each pathway using p < 0.05.

## Results

We delineated 93 unique observation units (average size = 0.2 km^2^, min = 0.01 km^2^, max = 1.6 km^2^, total = 21.5 km^2^) and summarized riparian productivity, grazing information, climate, and wildfire for each over 31 years, representing 2,883 total observations. NDVI ranged from 0.10 to 0.70 across all observation units and years; observation units with conventional grazing had average peak NDVI values of 0.27, while units with exclosure or conservation-oriented grazing averaged 0.35 and 0.34, respectively. Of the total observations, we used 541 for the statistical analysis: these observations occurred during the 13 years where high-resolution aerial imagery was available for identifying beaver dams; each watershed had 5 to 7 years of high-resolution imagery available with an average lag of 4.5 years between observations (min. = 2, max. = 12; Table 2). By watershed, the final available observations total 106 for 6 years in South Fork Salmon Falls Creek, 105 for 5 years in Jakes Creek, 84 for 7 years in Susie Creek, 168 for 6 years in Whitehorse Creek, and 78 for 6 years in for Willow Creek.

We identified 1,510 beaver dams in 27 aerial imagery datasets. These dams occurred at 1,083 unique locations, and 28% of locations had persistent dams which occurred in multiple imagery sets. Multiple dams in multiple watersheds appear to have persisted for over 15 years. The greatest density of beaver dams identified within an observation unit was 10 dams on 0.47 km of stream within an exclosure on Willow Creek in 2009. The largest number of dams observed in a watershed within a single year was 245 on 59.5 km of stream in Susie Creek in 2015. The number of dams increased in all watersheds between the first and last imagery dates (Table 2).

Wildfire occurred in four watersheds and included the Camp Creek Fire of 2000 in South Fork Salmon Falls Creek, the Basco and Suzie Fires of 2006 and Camp Creek fire of 2007 in Susie Creek, and the Holloway Fire of 2012 in Willow and Whitehorse Creeks. We attributed all observation units as burned for the year of the fire and the following year except for those within the Holloway Fire boundary, which burned in August after pre-fire satellite imagery had been collected and were attributed as burned for the 2 years following the fire. Limited availability of aerial images for characterizing beaver dam densities constrained our evaluation of wildfire effects to the Susie, Willow, and Whitehorse Creek watersheds.

**Fig 2:**
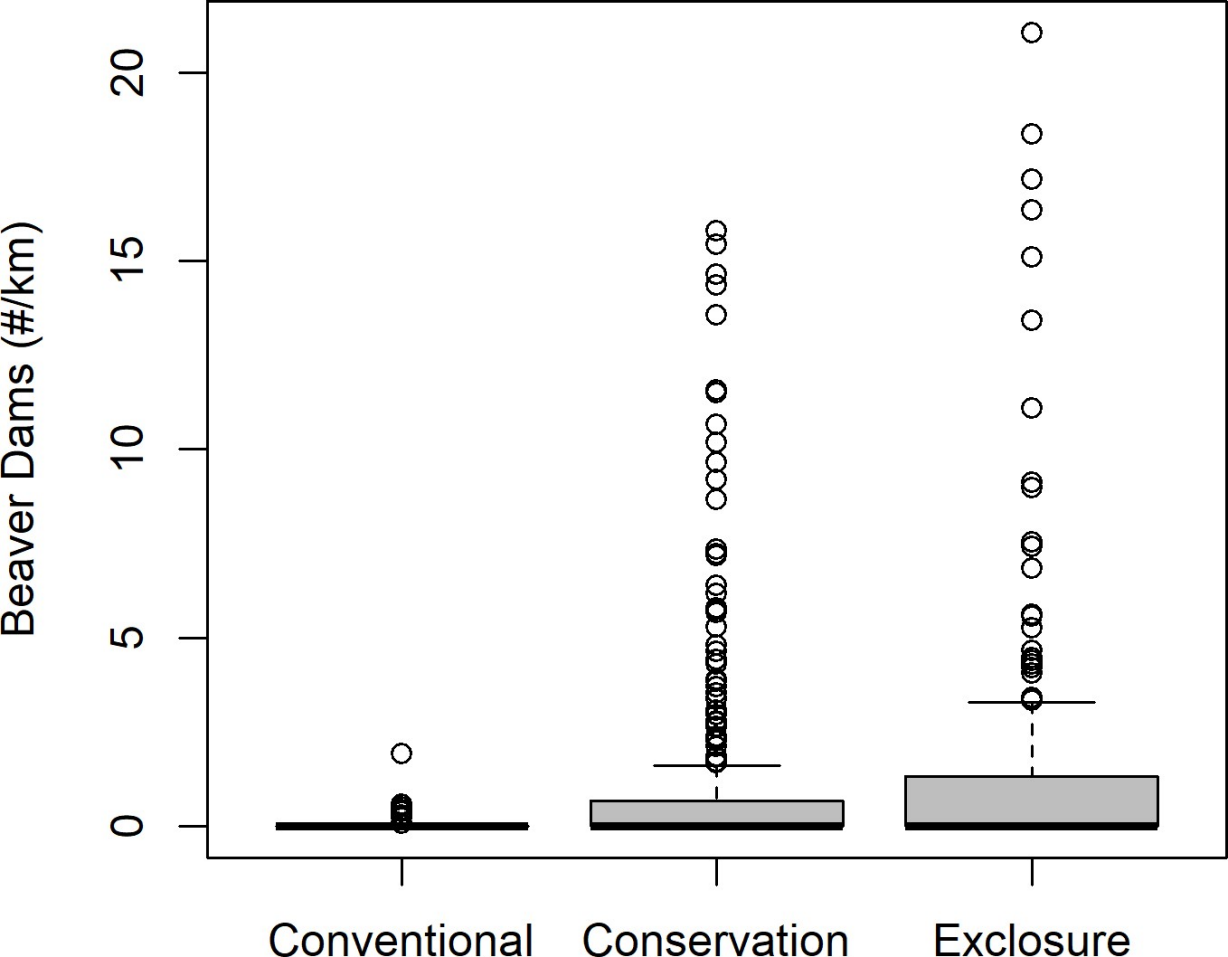
Beaver dam density by grazing treatment.

As we hypothesized, the linear mixed models revealed a positive relationship between riparian productivity and conservation-oriented grazing or exclosure, beaver dam density, precipitation, and stream size, and a negative relationship between riparian productivity and stream slope and recent wildfire. Model selection showed only four plausible models (ΔAIC_c_ ≤ 4; Table 3). The strongest Pearson correlation among candidate spatial covariates was *r* = 0.604 between precipitation and EDDI, which were never included in the same candidate models; this suggests that multicollinearity was not an issue in any candidate model. Of all variables evaluated, EDDI was not in any plausible model, and stream size and the beaver x grazing interaction were each in only two of the top four plausible models (Table 3). We interpreted the most plausible candidate model in the top set without the beaver x grazing interaction term (third most plausible model) because the interaction term had little effect on the log-likelihood and appeared to be uninformative [95], exploratory analysis showed large uncertainty in parameter estimates for interaction terms (S1 Fig), and there were very few beaver dams under conventional grazing (Fig 2). The interpreted model fit the data well (marginal R^2^ = 0.29; conditional R^2^ = 0.83).

**Table 3:** Candidate models explaining how spatial covariates influence riparian vegetation productivity (NDVI).

Candidate models	df	logLik	AICc	ΔAICc	w_i_
BeaverxSlope +BeaverxGraze + Elev + Fire + Size + Precip	14	-365.7	760.15	0.0	0.38
BeaverxSlope +BeaverxGraze + Elev + Fire + Precip	13	-367.0	760.67	0.5	0.29
BeaverxSlope + Graze + Elev + Fire + Size + Precip	12	-368.6	761.71	1.6	0.18
BeaverxSlope + Graze + Elev + Fire + Precip	11	-370.1	762.63	2.5	0.11
BeaverxSlope +BeaverxGraze + Elev + Fire + Size + Precip	13	-369.6	765.96	5.8	0.02
BeaverxSlope +BeaverxGraze + Elev + Fire + Precip	12	-370.9	766.32	6.2	0.02
Beaver + Slope +BeaverxGraze + Elev + Fire + Size + Precip	11	-374.6	771.71	11.6	0.00
Beaver + Slope +BeaverxGraze + Elev + Fire + Precip	10	-376.1	772.57	12.4	0.00

Only models with ΔAICc < 15 are shown, and all models with an interaction term also had main effect terms. df = degrees of freedom; logLik = Log-likelihood; wi = Akaike weights

Standardized parameter estimates from the fitted model showed that conservation-oriented grazing and exclosures had similar positive effects on NDVI (Table 4). NDVI was significantly higher under conservation-oriented grazing and exclosures vs. conventional grazing (p < 0.001; Table 4), but there was no significant difference between the effect of conservation-oriented grazing and exclosure on NDVI (*t* = 0.841; p = 0.401). Beaver had a positive effect on NDVI but only on low-gradient streams, as indicated by the interaction between beaver dam density and stream slope. While the effect of beaver was independent of grazing treatment in the interpreted model, there were very few beaver dams under conventional grazing (Fig 2). The un-standardized parameter estimates provide a means to characterize the gains in riparian productivity in our study watersheds created by conservation grazing and beaver management relative to sites with conventional grazing and without beaver. Our models indicate an expected increase in NDVI of 25% (± 14% 2 SE) with livestock exclosures and high beaver dam densities (i.e., 2 beaver dams/km) and 20% (± 7% 2 SE) with conservation-oriented grazing and high beaver dam densities, based on an average NDVI of 0.27 across the 31-year period for all observations units with conventional grazing and no beaver present.

**Table 4:** Parameter estimates and p-values from a multiple regression model predicting NDVI.

	Un-standardized		Standardized		
Parameter	*b*_i_	2 SE	*b*_i_	2 SE	p-value
Intercept	-0.149	0.175	-0.174	0.247	0.158
Precipitation (dm)	0.021	0.003	0.262	0.038	<0.001
Fire (Unburned = 0; Burned = 1)	-0.039	0.014	-0.372	0.132	<0.001
Elevation (km)	0.214	0.093	0.389	0.169	<0.001
Grazing: Conservation (Conventional = 0; Conservation = 1)	0.030	0.013	0.282	0.123	<0.001
Grazing: Exclosure (Conventional = 0; Exclosure = 1)	0.043	0.032	0.403	0.302	0.008
Stream size (km^2^)	0.021	0.024	0.097	0.111	0.082
Beaver dam density (#/km)	0.012	0.003	0.146	0.061	<0.001
Slope (%)	-0.012	0.007	-0.267	0.117	<0.001
Slope x Beaver	-0.003	0.002	-0.146	0.083	<0.001

Standardized parameter estimates are scaled and centered. 2 SE = 2 standard errors.

**Fig 3:**
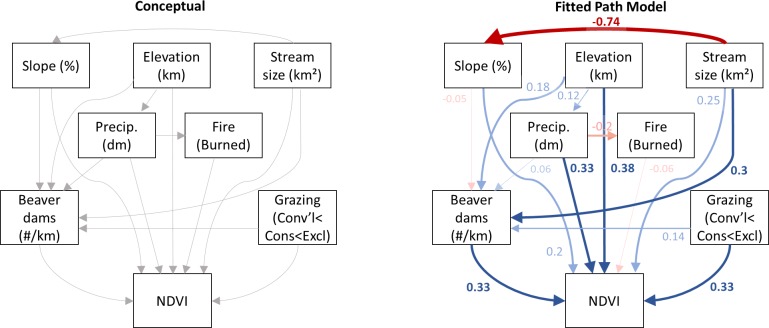
Conceptual and fitted path models. Fitted path model only shows effects with p < 0.05. Blue lines in the fitted model indicate positive effects, and red lines indicate negative effects. Values indicate standardized path coefficients; values around 0.3 are interpreted as moderate effects, while values around 0.1 are interpreted as weak effects.

Path analysis showed direct and indirect linkages between riparian productivity, grazing management, beaver density, climate, and stream characteristics in both shared and contrasting ways with the linear mixed modelling approach. Model fit was acceptable based on the comparative fit index (cfi = 0.954; root mean squared error approximation = 0.081). The effect of slope on beaver dam density (p = 0.282) and wildfire on NDVI (p = 0.083) were non-significant at α = 0.05. The fitted model demonstrated a direct effect of high water year precipitation, high beaver dam densities, and high elevations on high riparian productivity (Fig 3). High beaver dam densities are associated with larger streams, confirming the mediating effect of stream characteristics on beaver dam density. Conservation grazing approaches have moderate direct and weak indirect effects on high riparian productivity; indirect effects occur as grazing management is linked to increased beaver dam densities, which directly affects riparian productivity. Wildfire is associated with lower water year precipitation amounts. Large stream size is also directly linked to high riparian productivity; however, the linkage is weak.

**Fig 4:**
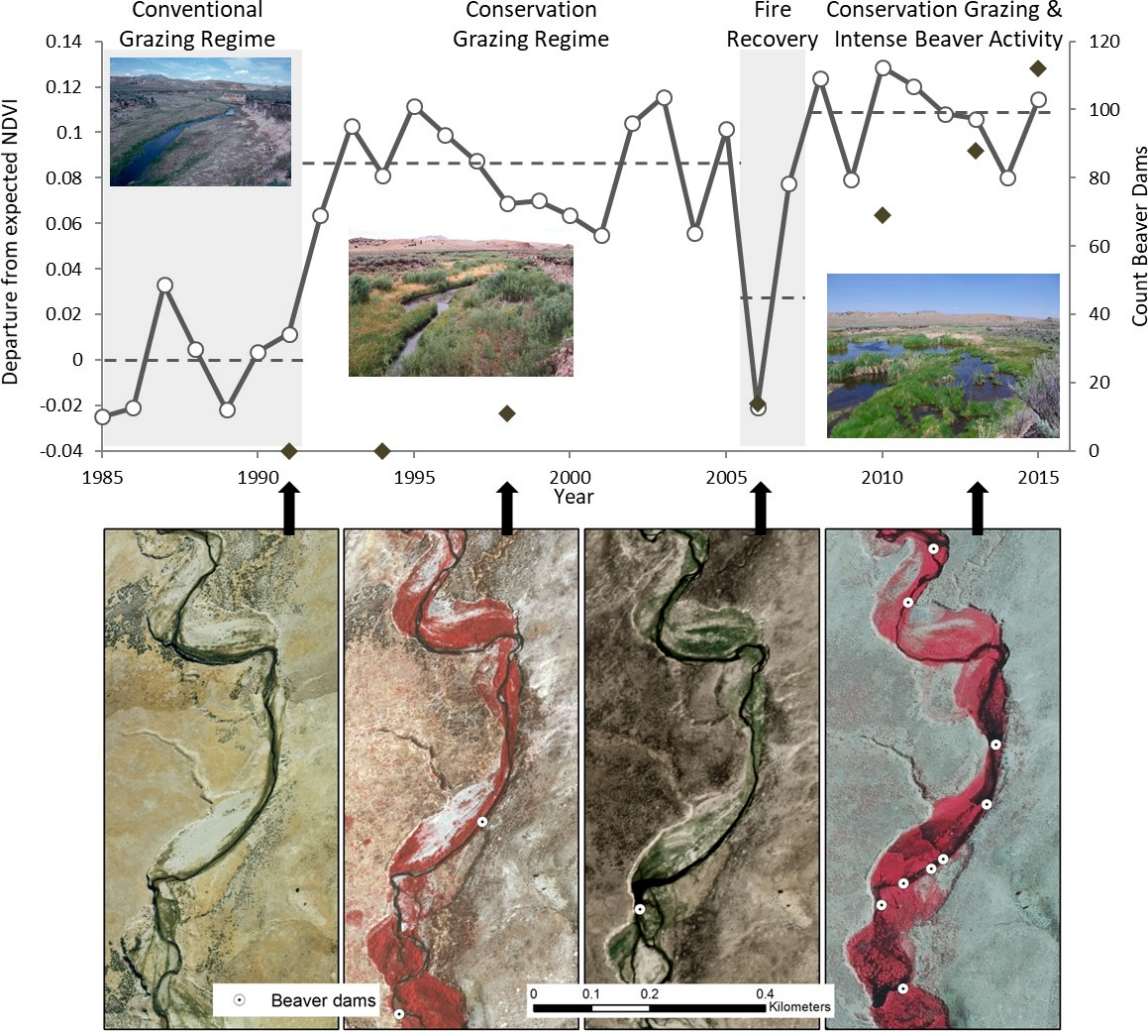
Time series of riparian vegetation recovery. Top panel: Departure from expected NDVI under conventional grazing regimes and 0 beaver (solid line, left y-axis) and beaver dam count (diamonds, right y-axis) time series data for the Susie Creek riparian pasture in the Carlin Field allotment. Departure from expected NDVI by year is represented using each year’s residual from the linear relationship between NDVI and water year precipitation for Susie Creek watershed observation units with conventional grazing regime, no wildfire effects, and 0 beaver dam observations. Negative departure values indicate lower than expected NDVI values given that year’s precipitation; positive values indicate higher than expected NDVI. Average NDVI departure for 4 treatment and condition periods shown with dashed lines. Bottom panel: Example aerial photos from a portion of the pasture from 1991, 1998, 2006, and 2013 showing riparian vegetation extent and beaver dams. Aerial photos are reprinted from USDA Farm Service Agency and site photographs are reprinted from US Bureau of Land Management, both under a CC BY license (public domain).

Fig 4 demonstrates the generalized progression of recovery in the systems we studied using a 3-decade riparian area NDVI time series for a single Susie Creek pasture. First, removal or reduction in grazing pressure during the growing season allows for the rapid establishment or recovery of woody streamside vegetation within 3 years. Beaver then colonize or expand their habitat use to take advantage of the woody vegetation, especially willow. As beaver populations increase, additional dam building activity enhances floodplain inundation, facilitating additional riparian vegetation productivity. Wildfire can reduce this recovery, but the effects are only temporary.

## Discussion

Multiple sensitive taxa depend on riparian areas within the Great Basin, and land managers are increasingly using livestock grazing management and restoration techniques involving beaver reintroduction or beaver dam analogues to improve riparian area condition and function [28, 36–39]. Response of riparian area vegetation to these treatments can occur at variable rates and can be influenced by disturbances such as drought and wildfire, making it difficult to parse out the effects of management actions without long-term evaluation of responses. The ability to apply remote sensing for effective long-term monitoring can improve our understanding of the interactions between grazing management and beaver and help land managers to better plan and evaluate their restoration actions within riparian areas.

Prior research has elucidated relationships between grazing management, beaver, climate, and stream characteristics individually or in tandem relative to riparian area productivity in arid and semi-arid landscapes. For example, Huntington and others [48] described the correlation between climate and riparian vegetation vigor measured as NDVI, while multiple studies have demonstrated recovery of willow and other riparian vegetation with exclosure or alternative management of livestock grazing [73, 96–99], including in adjacent watersheds [53, 100, 101]. Similarly, an increasing body of literature describes the anticipated riparian area benefits associated with beaver activity and identifies those physical and biological site conditions conducive to extensive beaver dam development [37–47, 87]. While others have described the potential mechanisms through which grazing can limit beaver and riparian recovery [77, 88, 102], our results are unique in demonstrating that grazing management which emphasizes riparian recovery objectives can be an important precursor to beaver activity, and that the productivity and vigor of riparian vegetation is amplified where these grazing approaches and beaver activity occur simultaneously.

Numerous studies suggest that the warming and drying effects of changing climate will cause riparian and aquatic organisms to shift their distributions to higher elevations [103–105], and that droughts are expected to increase in duration and severity as the climate continues to warm [106]. Riparian habitats are widely regarded as having high inherent resiliency to changing climate by virtue of their linear connectivity, elevated moisture levels, and microclimate [107–109]. By evaluating our model’s un-standardized parameter estimates for grazing treatments and beaver densities relative to those for annual precipitation and elevation (Table 4), we demonstrate that beaver management and conservation-oriented grazing or exclosures can enhance the resiliency of riparian ecosystems. The average increase in NDVI achieved by exclosure and high beaver dam densities is equivalent to moving a typical site in our study up in elevation by 314 m or increasing typical water year precipitation by 316 mm, nearly half of the range of precipitation observed across all sites and years in our study. Under conservation-oriented grazing and high beaver dam densities, the equivalent gains are 254 m of elevation or 256 mm of precipitation. Grazing management which emphasizes riparian recovery objectives can therefore not only increase riparian habitat for wildlife and fish but may also partially mitigate climate warming effects and increase resiliency to climate variability.

We report beaver dam densities comparable to other dryland systems [41], but document multiple examples of beaver dams persisting at least 15 years, over twice the previously reported duration for the region [44]. The beaver dam densities we evaluated likely represent an underestimation of the number of dams present in the systems. Research in nearby portions of Oregon has described detection issues related to using aerial imagery for identifying beaver dam locations, although the issues are less pronounced in arid landscapes [54].

We confirm that immutable stream characteristics such as stream size, slope, and elevation can dictate riparian area productivity, but the effects of disturbance or restoration and recovery are also often determined by other site characteristics. For example, wildfire effects are often heterogeneous in riparian systems due to variability in fuel loads, fuel type, topographic position, and microclimate dynamics [78], which may explain why wildfire is important in our linear mixed models, but a weak predictor in the path model. Site characteristics such as soil type may further dictate the rate of riparian area recovery following changes in grazing regime [35] or cause variable responses [73], and the initial rate of vegetation recovery may be controlled by climatic conditions [110]. While grazing, wildfire, and climate represent important stressors within Great Basin riparian systems, there are other potential sources of riparian area disturbance, including altered streamflow regimes, invasive plants, and agricultural water use. These disturbances may affect the physical form of floodplains, the composition of the vegetation community, water availability, and the corresponding potential for riparian vegetation recovery and restoration [19, 111, 112].

We demonstrate that grazing management within exclosures can yield riparian area vegetation productivity comparable to those produced by conservation-oriented grazing management, and that beaver dams can increase the recovery effects of each of these grazing strategies, but other considerations may dictate which treatment land managers apply to meet riparian objectives. In practice, conservation-oriented grazing strategies may be preferable to exclosures due to ease of implementation, flexibility, and scale of impact [28, 100]. We acknowledge that our categorization of grazing management into three treatments may be overly simplistic but chose general categories to facilitate comparison across watersheds and time. Similarly, management prescriptions may not correspond with actual use; exclosures, for example, can be subject to occasional or frequent livestock use due to lack of maintenance [73].

Monitoring using satellite-derived vegetation indices may not adequately capture specific habitat attributes which are associated with livestock use, meaningful to fish and wildlife, and commonly used in grazing management prescriptions. For example, field-based indicators of grazing pressure such as decreased stubble height, increased stream bank alteration, and decreased riparian vegetation cover have been linked to decreases in habitat quality for salmonid fishes [32, 34, 113, 114]. An important next step for monitoring with remote sensing is linking the response of conservation targets (i.e., rare taxa) or their specific habitat requirements to factors like NDVI, which can now be easily monitored using historical data archives and cloud computing.

Continued monitoring of our sites may reveal other restoration trajectories associated with grazing management and beaver as incised stream channels aggrade and fill behind beaver ponds [38], and help characterize the time required for additional recovery of riparian vegetation; low-gradient, larger streams in all study watersheds have inset channels below the historical floodplain and full recovery of potential vegetation at sites may require in excess of 5 decades [115]. For example, from 2016–2018 the Susie Creek pasture in Fig 4 has experienced beaver dam failure or abandonment as vegetation communities shifted from woody to emergent species (e.g., cattails, *Typha latifolia*), followed by early evidence of recolonization of willow within the elevated floodplain and the potential for a restart of the beaver colonization cycle. Long-term analyses of grazing treatments such as ours can be challenging due to the difficulty in tracking down historical records of grazing management, although recent efforts to document vegetation restoration treatments [116] and beaver-related restoration [117] do exist in the Great Basin.

Restoration with conservation grazing approaches and/or beaver management represents a low-effort and low-expense strategy for addressing causes of degradation using natural processes which can be scaled to broad spatial extents. Such strategies are increasingly valued relative to more traditional channel-based, active restoration approaches [118, 119]. As the result of those grazing management actions and compounded by the effect of natural beaver colonization, we demonstrate potential increases in riparian area productivity ranging up to 39% at sites with conservation-oriented grazing or exclosures and high beaver activity. These habitats represent an important mesic resource for fish and wildlife which is rare within the overall landscape, scarce on public lands in the ecoregion [18], and valued as a key resilient habitat in the face of changing climate [107–109].

## Supporting information

S1 Dataset Data used for analyses. (XLSX)

S1 Fig Predicted effect of beaver density on NDVI under three grazing treatments. Model selection results suggested that the most-plausible linear mixed model explaining variation in NDVI as a function of covariates included a grazing treatment x beaver density interaction term (Table 3). However, exploration of this top model revealed wide confidence intervals for the effect of beaver density under a conventional grazing regime. Further exploratory analysis of the data showed that beaver rarely occurred when under a Typical grazing regime, yielding much uncertainty in the parameter estimate and an unknown influence on model behavior. We therefore did not consider nor interpret models with the interaction term.
(TIF)
