# Supplementary figures and images for: Livestock management, beaver, and climate influences on riparian vegetation in a semi-arid landscape
Source: PLoS One. 2018 Dec 11;13(12):e0208928. doi: 10.1371/journal.pone.0208928 (PMC6289506; doi:10.1371/journal.pone.0208928)

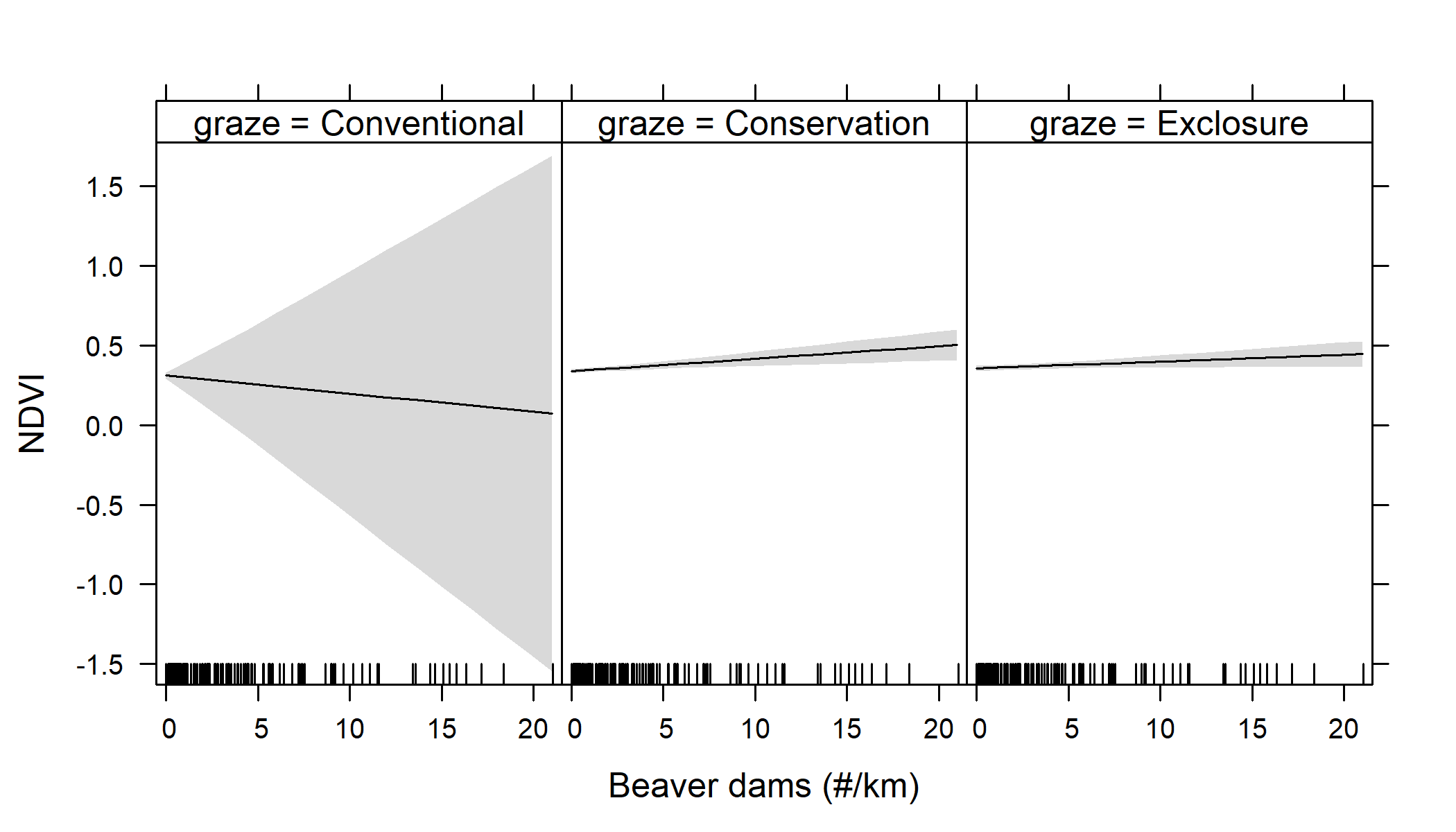

Supplement: S1 Fig — Model selection results suggested that the most-plausible linear mixed model explaining variation in NDVI as a function of covariates included a grazing treatment x beaver density interaction term (Table 3). However, exploration of this top model revealed wide confidence intervals for the effect of beaver density under a conventional grazing regime. Further exploratory analysis of the data showed that beaver rarely occurred when under a Typical grazing regime, yielding much uncertainty in the parameter estimate and an unknown influence on model behavior. We therefore did not consider nor interpret models with the interaction term. (TIF) [file pone.0208928.s002.tif]
